# The Role of Intermittent Fasting in the Activation of Autophagy Processes in the Context of Cancer Diseases

**DOI:** 10.3390/ijms26104742

**Published:** 2025-05-15

**Authors:** Waleria Wolska, Izabela Gutowska, Agata Wszołek, Wojciech Żwierełło

**Affiliations:** 1 Department of Medical Chemistry, Pomeranian Medical University in Szczecin, Powstańców Wlkp. 72, 70-111 Szczecin, Poland; volskayv@gmail.com (W.W.); wojciech.zwierello@pum.edu.pl (W.Ż.); 2 Department of Clinical and Molecular Medicine, Norwegian University of Science and Technology, Erling Skjalgssons gate 1, 7030 Trondheim, Norway; 3 Institute of Biology, University of Szczecin, Wąska 13, 71-415 Szczecin, Poland; agata.wszolek@usz.edu.pl

**Keywords:** autophagy, cancer, diets, calorie restriction, intermittent fasting

## Abstract

Intermittent fasting (IF) is a dietary approach that influences key metabolic pathways, including autophagy—a crucial mechanism in maintaining cellular homeostasis. Autophagy plays a dual role in oncogenesis, acting both as a tumor suppressor and a survival mechanism under metabolic stress. IF has shown potential for reducing cancer risk and enhancing therapeutic efficacy by sensitizing tumor cells to chemotherapy and radiotherapy. However, its effects depend heavily on the type and stage of cancer. Potential risks, such as excessive weight loss and malnutrition, require careful evaluation. Further clinical studies are needed to optimize IF protocols as adjuncts to cancer therapy. This review discusses autophagy mechanisms induced by IF, their therapeutic implications in oncology, and the limitations of this dietary strategy.

## 1. Introduction

Cancer continues to be one of the most pressing global health and socio-economic challenges. In 2022, the World Health Organization (WHO) reported over 19 million new cancer cases and nearly 10 million cancer-related deaths, making it the second leading cause of death worldwide. Alarmingly, projections indicate that the incidence of cancer will increase by over 70% by 2050, driven primarily by aging populations, lifestyle changes, and environmental factors. In low- and middle-income countries, limited access to diagnostic tools and treatment options worsens survival disparities, while in high-income countries, cancer continues to place an escalating burden on healthcare systems. International initiatives like the United States’ Cancer Moonshot and Europe’s Beating Cancer Plan focus on prevention, early detection, and the development of more effective therapies, highlighting the pivotal role of scientific research in the fight against cancer [1,2]. On a global scale, the increasing incidence of malignant tumors necessitates the search for innovative therapeutic strategies based on a precise understanding of the molecular processes underlying neoplastic transformation [3].

A deeper understanding of cancer biology—particularly the roles of autophagy and metabolic dysregulation—is critical for identifying new therapeutic targets and improving treatment outcomes. Autophagy, as an intracellular recycling process, plays an ambiguous role in carcinogenesis, being able to both support cancer cell survival under metabolic stress and induce their death under appropriate therapeutic conditions. In turn, metabolic reprogramming—including the Warburg effect—enables cancer cells to maintain rapid proliferation and resistance to treatment. These processes not only influence cancer cell survival and resistance to treatment but also interact with systemic metabolic states, making them important avenues for both pharmacological and dietary interventions [4]. Lung cancer remains the most commonly diagnosed malignancy in both women and men. Its high mortality is attributed not only to tobacco use and electronic cigarette consumption but also to increasing environmental pollution, including smog [5]. Given the rising incidence of this disease, there is an urgent need to explore more effective and less invasive therapeutic approaches. One promising supportive strategy in cancer treatment is intermittent fasting (IF), which may enhance therapeutic efficacy while mitigating the adverse side effects of chemotherapy and radiotherapy [6].

Fasting has been practiced for centuries across various cultures, primarily for religious purposes, as seen in Christian Lent and Islamic Ramadan. Contemporary research on IF focuses on its impact on metabolic health and its potential therapeutic applications, including oncology. The beneficial effect of IF is associated with, among others, the improvement of cancer cell sensitivity to treatment and the modulation of the tumor metabolic environment [7]. Therefore, the aim of this paper is to present the current state of knowledge regarding the effects of different IF models on the activation of autophagy in cancer-related conditions.

## 2. Autophagy—Key Mechanism of Cellular Homeostasis

Autophagy is a fundamental cellular process responsible for the degradation and recycling of intracellular components, including damaged organelles, misfolded proteins, and redundant cytoplasmic structures [8]. The term ‘autophagy’, derived from Greek, literally means ‘self-eating’, reflecting the essence of this process. It is essential for maintaining cellular homeostasis, eliminating metabolic waste, and providing energy substrates during stress conditions, such as nutrient deprivation. During IF, nutrient-sensing pathways respond to reduced energy availability by activating autophagy. Specifically, AMP-activated protein kinase (AMPK) is upregulated, which in turn inhibits the mechanistic target of rapamycin complex 1 (mTORC1)—a major autophagy suppressor. This inhibition allows the activation of the UNC-51-like kinase 1 (ULK1) complex, initiating the autophagy cascade [9,10]. Through this mechanism, IF promotes cellular adaptation to metabolic stress and enhances the clearance of damaged cellular components [11].

### 2.1. Types of Autophagy

Three primary types of autophagy can be distinguished, each characterized by distinct mechanisms of intracellular degradation:

- Microautophagy involves the direct engulfment of small portions of the cytoplasm by lysosomes. This process facilitates nutrient recycling and supports cellular metabolic balance [12].
- Chaperone-mediated autophagy (CMA) is a highly selective mechanism in which chaperone proteins (e.g., Hsp70) target specific substrates to lysosomes via the lysosomal membrane protein LAMP2A. This type of autophagy plays a crucial role in the removal of misfolded proteins and the maintenance of proteostatic homeostasis [13].
- Macroautophagy is the most extensively studied and complex form of autophagy. It involves the encapsulation of damaged organelles and proteins within autophagosomal membranes, followed by their delivery to lysosomes for degradation. Macroautophagy is essential for cellular stress responses and intracellular resource recycling [14].

While each type of autophagy plays a distinct role in maintaining cellular homeostasis, their dysregulation can have profound implications for a variety of diseases, including neurodegenerative conditions, metabolic disorders, and cancer. Understanding these roles is essential for targeting autophagy as a therapeutic strategy.

### 2.2. Autophagy in Non-Cancerous Disease Processes

While autophagy is widely recognized for its dual role in cancer, it also plays a critical part in the pathogenesis of various non-neoplastic diseases. As a key regulator of cellular homeostasis, it facilitates the clearance of damaged proteins and organelles, supporting tissue function. Disruptions in this process are linked to neurodegenerative, cardiovascular, metabolic, and musculoskeletal disorders. Given its broad physiological relevance, autophagy modulation—e.g., through intermittent fasting—emerges as a potential therapeutic strategy outside of oncology [8,15,16].

Disruptions in the autophagic process play a crucial role in the pathogenesis of neurodegenerative diseases, which are marked by the accumulation of toxic protein aggregates. Autophagy is essential for the degradation and removal of pathological structures, impacting the progression of disorders such as Alzheimer’s and Parkinson’s diseases [17]. Animal model studies have shown that mice with neuron-specific ATG5 deficiency accumulate abnormal cytoplasmic proteins, leading to motor dysfunction and neurodegeneration [18]. ATG5 encodes a protein that forms a conjugate with ATG12, functioning as an E1-like activating enzyme within a ubiquitin-like system crucial for autophagosome formation. This protein participates in various cellular processes, including mitochondrial quality control following oxidative damage, suppression of the innate antiviral response, lymphocyte development and proliferation, MHC class II antigen presentation, adipocyte differentiation, and apoptosis. Multiple transcript variants of ATG5 exist, encoding different protein isoforms, which further diversify its functional roles [19]. However, current evidence does not clearly establish which specific ATG5 isoform is predominantly involved in AD. In Alzheimer’s disease, Tau proteins can damage lysosomal membranes, thereby activating autophagy as a protective mechanism. However, under pathological conditions, impaired fusion between autophagosomes and lysosomes may contribute to disease progression [20,21].

Autophagy also plays a crucial role in cardiovascular function. Due to their limited regenerative capacity, cardiomyocytes heavily rely on this process for the removal of damaged organelles and proteins. Studies have shown that excessive activation of the mTORC1 signaling pathway in the heart, which regulates cell growth and metabolism, impairs autophagy, thereby increasing the susceptibility of cardiac muscle to damage, particularly under ischemic conditions [22,23].

Disruptions in the autophagic process have a significant impact on the development of metabolic diseases, including type 2 diabetes, obesity, and non-alcoholic fatty liver disease (NAFLD). Autophagy is crucial for maintaining insulin homeostasis and regulating lipid and glucose metabolism. The dysregulation of genes involved in autophagy, such as ATG7 and Becn2, has been shown to lead to insulin resistance and the deterioration of metabolic parameters [24,25]. In obesity, autophagy plays a role in adipogenesis. Its inhibition leads to a reduction in adipose tissue mass and increases the expression of the protein UCP1, promoting the conversion of white adipose tissue into its more metabolically active form—brown adipose tissue [26].

Autophagy is a critical element in the homeostasis of the musculoskeletal system, regulating the functions of muscles and bones. Its dysregulation can lead to sarcopenia and muscle degeneration, as demonstrated in studies on mice with ATG7 gene deletion. These animals showed the accumulation of morphologically and functionally abnormal mitochondria in their cells, as well as muscle mass loss, underscoring the essential role of autophagy in maintaining proper muscle structure and function [27]. In the skeletal system, autophagy regulates the balance between osteoblasts and osteoclasts, playing a crucial role in bone mineralization and resorption processes [28].

### 2.3. Autophagy in Cancer Diseases

Cancer is a heterogeneous group of diseases characterized by uncontrolled cell growth and proliferation. Recent studies highlight cancer as one of the most significant challenges in modern medicine and public health [29]. The incidence of cancer is steadily rising worldwide, and effective treatment remains elusive due to cancer cells’ ability to invade surrounding tissues, form metastases, and develop resistance to multiple drugs [30]. Tumorigenesis is a multi-step process, driven by both genetic predispositions and environmental factors, leading to the accumulation of mutations and disruptions in mechanisms controlling the cell cycle, apoptosis, and autophagy [31]. The dysregulation of these processes promotes tumor progression, enhances resistance to treatment, and contributes to the emergence of an aggressive phenotype [29].

Autophagy plays a key role in cancer prevention by facilitating the degradation of damaged organelles and proteins, thereby preventing their accumulation within cells. However, mutations in essential genes regulating autophagy, such as Ambra1 [32], ATG5 [33], and ATG7 [34], may promote the onset of tumorigenesis by impairing the mechanisms that eliminate damaged cells. An example of this process is melanoma, where low expression of ATG5 correlates with poorer prognosis in patients [35].

Studies using animal models have demonstrated that deleting the ATG7 gene accelerates melanogenesis in the skin, accompanied by an increase in hyperactive BRAF expression, which promotes tumor development [36]. Interestingly, the use of autophagy inhibitors under specific conditions reduces the risk of tumor recurrence, although monitoring for the emergence of premalignant changes is required. For instance, in cases where a tumor is highly dependent on autophagy as a survival mechanism for cancer cells (e.g., under conditions of nutrient deprivation or oxidative stress), its inhibition can lead to increased cancer cell death and reduced risk of recurrence. On the other hand, prolonged inhibition of autophagy in healthy tissues may promote abnormal cell proliferation and the formation of polyps [37]. Autophagy also acts preventively by selectively removing damaged organelles, regulating mitophagy, and eliminating cells with DNA damage, thereby reducing the risk of neoplastic transformation [37]. Additionally, the role of chaperone-mediated autophagy (CMA) is emphasized, as the proper functioning of this mechanism is crucial for protection against cancer. Its action is based on the degradation of proteins that regulate cell proliferation, such as MYC, TPT1, and MDM2 [38,39,40]. Mice with impaired CMA function in hepatocytes showed an increased frequency of neoplastic changes in the liver [41]. Furthermore, autophagy exhibits anti-inflammatory effects by regulating reactive oxygen species (ROS) levels and participating in the elimination of damaged mitochondria through mitophagy, which reduce pro-carcinogenic oxidative stress [42,43].

#### Autophagy as a Mechanism Supporting Cancer Progression

While autophagy serves a protective function during the early stages of tumorigenesis, in advanced stages of the disease, it may promote tumor progression. Cancer cells, particularly under conditions of nutrient deprivation and hypoxia, can exploit autophagy to survive and adapt to the unfavorable conditions of the tumor microenvironment [44]. Increased autophagic activity is observed in many cancers as a compensatory mechanism, providing tumor cells with essential nutrients through the degradation of their own structures. Consequently, inhibiting autophagy in the early stages of cancer development may potentially hinder further tumor progression [44].

Studies have shown that solid tumors exhibit elevated chaperone-mediated autophagy (CMA) activity, which supports their survival and growth [45]. This mechanism is linked to the selective degradation of tumor-suppressor proteins, such as RND3 (Rho family of GTPases) [46] and MCL1 (myeloid cell leukemia-1) [47]. These findings are being used to develop new therapeutic strategies that involve the selective inhibition of CMA to limit tumor growth [48], while simultaneously supporting autophagic processes in healthy cells [49].

As previously mentioned, the role of autophagy in tumorigenesis is ambiguous—on one hand, it can inhibit tumor initiation, but on the other hand, it supports tumor adaptation and survival under metabolic stress conditions [50]. For example, the BECN1 gene, which is crucial in the process of autophagy, undergoes deletion in ovarian, testicular, and breast cancers, suggesting its tumor-suppressive function [51]. Animal models have shown that low levels of autophagic activation promote the development of liver and lung tumors [52], and the deletion of ATG5 leads to the development of benign liver tumors [53]. Autophagy plays a protective role by counteracting oxidative stress and DNA damage, but in advanced cancers, it can provide cancer cells with resources enabling their survival and proliferation [54,55]. Furthermore, studies on breast cancer indicate that blocking autophagy through the inhibition of ATG5 and ATG7 limits migration and the ability of cancer cells to form metastases, suggesting the therapeutic potential of targeted inhibition of this process [50].

### 2.4. Pharmacological Modulation of Autophagy in Cancer Therapy

Autophagy can play both tumor-suppressive and tumor-promoting roles, making its regulation an important therapeutic target in cancer treatment. Therefore, research on autophagy focuses on its pharmacological modulation as a potential strategy for the treatment of malignant tumors [44,56,57].

#### 2.4.1. Autophagy Inhibition as a Therapeutic Strategy

Inhibiting autophagy can enhance the effectiveness of cancer therapies. It has been shown that combining autophagy inhibitors, such as chloroquine, bafilomycin, or 3-methyladenine, with chemotherapeutics increases their cytotoxicity [58]. For example, combining selumetinib and cytarabine with autophagy inhibitors significantly enhances therapeutic efficacy in colorectal cancers, while autophagy induction through rapamycin promotes cancer cell survival [59]. Mechanistically, these inhibitors block the late stages of autophagy, particularly the fusion of autophagosomes with lysosomes (chloroquine and bafilomycin) [60,61], or inhibit class III PI3K activity to prevent autophagosome formation (3-MA), thereby disrupting the recycling of damaged cellular components [62]. Similar effects were observed in melanoma, where autophagy inhibition using BPC11 (Biphosphinic Paladacycle Complex 11) increased the number of apoptotic cells, suggesting that autophagy acts as a protective mechanism for melanoma cells against treatment [63]. In melanoma cells, autophagy inhibition enhances ER stress and ROS accumulation, shifting the balance toward apoptosis [64,65]. Inhibition of autophagy may also increase the sensitivity of cancer cells to radiotherapy [66]. In ovarian cancer, the use of the autophagy inhibitor 3-methyladenine (3-MA) significantly enhanced the radiation effect, while the inhibition of apoptosis had no impact on cell survival [67]. Similar results were obtained in esophageal squamous cell carcinoma, where 3-MA caused cell cycle arrest in the G2/M phase, increasing the cells’ susceptibility to radiation [67,68]. This radiosensitizing effect is linked to impairments in DNA damage repair pathways in autophagy-deficient cells [69]. Moreover, autophagy plays a crucial role in tumor angiogenesis, the formation of new blood vessels that nourish tumors. Autophagy inhibition leads to decreased expression of VEGF (vascular endothelial growth factor), which may limit tumor vascularization and its progression [67,70]. This effect is mediated via the downregulation of the HIF-1α/VEGF axis, which is stabilized under hypoxic conditions in tumors but depends on autophagic flux to maintain metabolic adaptation [71].

#### 2.4.2. The Induction of Autophagy as a Therapeutic Strategy

Some therapies utilize autophagy induction as a therapeutic mechanism to induce cancer cell death. Rapamycin (sirolimus) and its analogs, such as Everolimus and Temsirolimus, are used as mTOR (mammalian target of rapamycin) inhibitors in cancer treatment [72]. Everolimus has been shown to be effective in inhibiting the growth of breast and kidney cancers through the induction of autophagy and the limitation of cancer cell proliferation [73]. mTORC1 normally suppresses autophagy by phosphorylating and inhibiting the ULK1 complex; its inhibition by rapamycin allows ULK1 activation and the initiation of autophagosome formation [74]. Metformin, known as an anti-diabetic drug, exhibits anti-cancer effects through the activation of AMPK (5′AMP-activated protein kinase) and autophagy induction. It has been shown that metformin can enhance the effectiveness of chemotherapy in pancreatic cancer and colorectal cancers [75,76]. AMPK activation under metabolic stress directly phosphorylates ULK1 and inhibits mTORC1 activity, promoting autophagy initiation. This dual control allows cells to adapt to energy deficits and influences therapy response depending on cellular context and p53 status [77]. Additionally, tricyclic antidepressants, such as imipramine, in combination with ticlopidine, also induce autophagy and lead to the death of glioblastoma cells through increased cAMP levels [78]. The cAMP-PKA pathway can modulate autophagy, but its role appears to be highly context-dependent, sometimes inhibiting autophagy initiation through ULK1 phosphorylation [79], while in glioblastoma models, it enhances ER stress-mediated autophagy [80]. Natural compounds such as resveratrol and spermidine have also been observed to promote autophagy. In the case of resveratrol, this occurs through the activation of the AMPK pathway and simultaneous inhibition of the mTOR pathway, which enhances the effects of chemotherapeutic drugs [81]. The effect of resveratrol on autophagy is dose-dependent: at low concentrations (≤10 μM), it activates protective autophagy in normal cells, while higher concentrations (>50 μM) can induce autophagic cell death in cancer cells, particularly when combined with other treatments (combination with rapamycin). This effect was mediated through the NGFR-AMPK-mTOR signaling pathway [82]. Spermidine, on the other hand, exhibits a similar effect but independently of the mTOR pathway, distinguishing it from other substances. Nevertheless, it has a significant therapeutic effect, particularly in liver cancers [53]. Spermidine stimulates autophagy via epigenetic regulation of autophagy-related genes (e.g., ATG5, LC3), acting through the inhibition of histone acetyltransferases and induction of hypoacetylation at promoter regions, particularly relevant in hepatocellular carcinoma [83,84]. Mechanistically, spermidine inhibits the acetyltransferase EP300, leading to chromatin remodeling and increased expression of autophagy-related genes. This epigenetic mechanism appears to be more active in malignant cells, which rely heavily on autophagic flux for metabolic adaptation, thereby conferring selective toxicity. In contrast, spermidine supports cytoprotective autophagy in non-transformed cells, making it context-dependent and potentially safer for therapeutic use [85]. Additionally, a randomized, placebo-controlled trial in older adults demonstrated that oral spermidine supplementation improved biomarkers of autophagy without adverse effects, supporting its translational potential in aging and disease-prone populations [86].

## 3. Intermittent Fasting

### 3.1. Types of Intermittent Fasting

**Figure 1:**
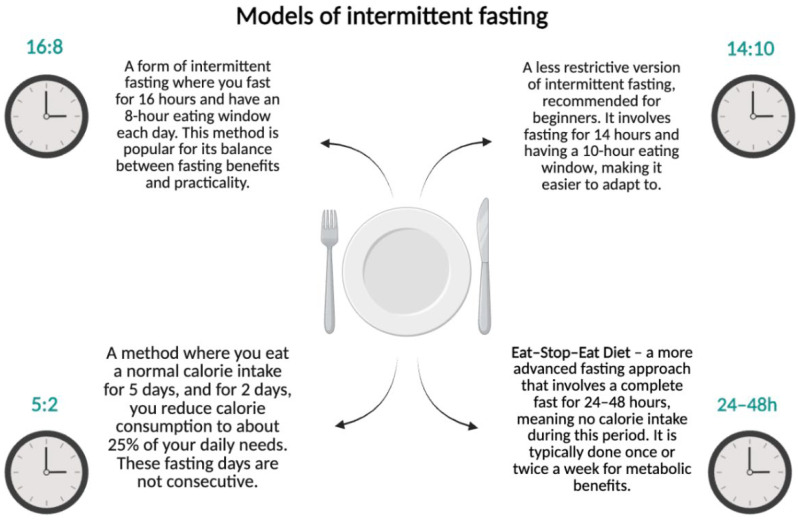
Models of intermittent fasting. Created in BioRender.com. (License number: GV285Q0XS3).

Intermittent fasting (IF) is a dietary regimen that alternates between periods of fasting and eating. Among the popular schemes, the following can be distinguished (Figure 1):

- 16:8—16 h of fasting with an 8 h eating window;
- 14:10—a less restrictive form recommended for beginners;
- 5:2—consuming a standard number of calories for 5 days and reducing intake to 25% of daily requirements for 2 days;
- Eat–Stop–Eat—complete fasting for 24–48 h.

The literature also includes other terms related to fasting, such as PF (periodic fasting), FMD (fasting-mimicking diet), and TRF (time-restricted feeding) [87,88,89,90].

### 3.2. The Impact of Intermittent Fasting on the Body

During IF, the body’s metabolism undergoes changes due to the limitation of nutrient availability. Studies indicate that IF can improve glucose tolerance, regulate insulin levels, and influence the function of the Langerhans islets in the pancreas, which is crucial for the prevention of type II diabetes [91]. Additionally, intermittent fasting may have neuroprotective effects by increasing neuronal resistance to oxidative stress and reducing levels of insulin-like growth factor (IGF-1), which may impact aging processes [17]. Research has shown that IF during Ramadan (approximately 18 h of fasting for 29–30 days) reduces LDL cholesterol levels and increases HDL cholesterol in women, as well as reducing inflammatory markers such as CRP and TNF-α [92,93]. Authors have identified autophagy activation as one of the key mechanisms underlying these effects, as it helps eliminate damaged cells and proteins, contributing to the improvement of the aforementioned processes [94].

Fasting-induced autophagy is triggered by reduced intracellular nutrient and energy levels, which activate AMPK and inhibit mTORC1—the major nutrient-sensing suppressor of autophagy. AMPK directly phosphorylates ULK1, initiating the formation of autophagosomes. In parallel, reduced insulin and IGF-1 signaling during fasting suppress the PI3K-Akt-mTOR pathway, further lifting the brake on autophagy [95,96].

Different fasting regimens can selectively engage distinct autophagy-related pathways. For example, short-term fasts (e.g., 16–24 h) primarily activate macroautophagy in metabolically active tissues such as the liver, muscle, and brain. This type of autophagy involves the sequestration of cytoplasmic material into double-membraned autophagosomes and is tightly regulated by the AMPK/mTOR/ULK1 axis [97]. In contrast, prolonged fasting (e.g., >48 h or multi-day fasts) also induces chaperone-mediated autophagy (CMA), which selectively degrades damaged or misfolded proteins via the LAMP-2A receptor recognition in lysosomes [98]. Selective mitophagy—the autophagic removal of damaged mitochondria—is also enhanced during prolonged fasts and is regulated by the PINK1–Parkin pathway [99].

### 3.3. Impact of Autophagy Induced by Intermittent Fasting on Cancer

**Figure 2:**
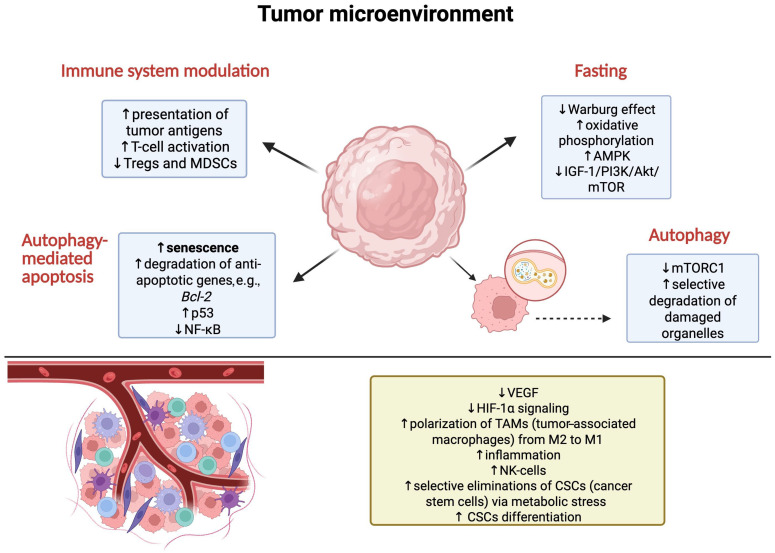
Mechanisms occurring under the influence of IF and autophagy in tumor cells and their microenvironment. Created in BioRender.com. (License number: UR285Q0RV4).

Intermittent fasting is gaining increasing interest as a potential strategy to support cancer therapy, mainly through its effects on autophagy and the metabolism of cancer cells. Although clinical studies in this area are limited, both in vitro and in vivo experiments suggest that IF can enhance the effectiveness of cancer treatment by regulating key metabolic and signaling pathways [100]. Intermittent fasting activates autophagy as an adaptive mechanism to nutrient deprivation, which may modulate tumor development and treatment [101]. It has been shown that fasting reduces the expression of glucose transporters GLUT1/2, which slow down cancer metabolism and increase the susceptibility of cancer cells to oxidative stress [102]. Furthermore, studies on cell and animal models have shown that intermittent fasting reduces glucose and insulin-like growth factor (IGF-1) levels [103], as well as insulin [104,105], resulting in the inhibition of the mTOR kinase pathway (PI3K/Akt/mTOR), suppression of mTORC1 [22], and activation of AMPK through increased ADP/ATP ratio in cells, which supports autophagy and induces apoptosis [103,104,105,106,107]. Moreover, IF counteracts the Warburg effect by promoting oxidative phosphorylation, leading to an increase in the production of reactive oxygen species (ROS) and enhanced oxidative stress in cancer cells [106,108], causing DNA damage and the activation of autophagy as a mechanism of cell death [100] (Figure 2).

Preclinical studies suggest that intermittent fasting (IF) may enhance the effectiveness of chemotherapy and targeted therapies by activating autophagy, regulating metabolic pathways, and modulating tumor immune surveillance. The mechanisms responsible for IF’s influence on autophagy primarily involve the inhibition of the mTOR pathway and the activation of AMPK [95]. The mTORC1 pathway is one of the main regulators of cellular metabolism, and its inhibition leads to autophagy induction, which may facilitate the elimination of cancer cells damaged by chemotherapy or radiotherapy [109,110]. In contrast, the activation of AMPK, which occurs in response to reduced nutrient availability during fasting, strengthens catabolic processes, including autophagy, supporting metabolic adaptation while simultaneously limiting tumor growth [106,111] (Figure 2).

In vitro and in vivo studies indicate that IF enhances the effectiveness of chemotherapy, including drugs such as cisplatin, cyclophosphamide, and doxorubicin [108,112,113]. The mechanisms underlying this synergy require further investigation, but it is known that IF affects the tumor microenvironment by reducing the polarization of M2 macrophages, which support tumor growth, while simultaneously strengthening the anti-tumor immune response [114]. Furthermore, fasting reduces the production of adenosine by cancer cells, inhibiting the activation of the JAK1/STAT pathway, thereby reducing cancer cell proliferation [115] (Figure 2).

Experiments on mouse models of fibrosarcoma have shown that chemotherapy (mitoxantrone or oxaliplatin) combined with 48 h fasting led to significant tumor growth inhibition. This effect was dependent on the presence of a functional immune system; no improvement in therapy efficacy was observed in mice lacking T lymphocytes, suggesting that IF may enhance immune surveillance dependent on autophagy [111]. Additionally, the beneficial effect of fasting disappeared in the case of autophagy deficiency resulting from reduced expression of the ATG5 gene, confirming the key role of autophagy in the synergistic action of IF and chemotherapy [116]. Furthermore, studies on mouse models have shown that in breast cancer, IF disrupts mitochondrial function, increasing the susceptibility of cancer cells to chemotherapeutic agents [117].

Experimental evidence suggests that intermittent fasting (IF) may enhance the effectiveness of chemotherapy, particularly in combination with drugs such as gemcitabine, which, when used in pancreatic cancer, leads to mTOR activation. It has been observed that IF can counteract this activation, enhancing the cytotoxic effect of the drug through autophagy and the degradation of key proteins associated with cell survival [118]. Studies in animal models and cell lines have confirmed that IF can reduce tumor size by modulating cancer metabolism and decreasing the availability of glucose and amino acids, which are essential for the rapid growth of cancer cells [114]. Limited human studies also suggest that dietary interventions, including fasting, may play a significant role in slowing the progression of various types of cancer (Table 1).

### 3.4. Potential Risks of Intermittent Fasting in Cancer Patients

Calorie restriction (CR) has been shown to have health benefits, reducing the risk of developing diseases, including cancer, and enhancing the effectiveness of chemotherapy and radiotherapy [103]. Due to the difficulties associated with long-term CR in cancer patients, intermittent fasting (IF) and ketogenic diets present alternative strategies that offer similar metabolic and therapeutic benefits [124]. Despite its potential benefits, IF may not be suitable for all cancer patients.

#### Risk of Weight Loss and Cachexia

Cachexia is a pathological condition characterized by significant weight loss and metabolic changes that can weaken cancer patients. It occurs in 50–80% of patients, particularly in those with pancreatic, esophageal, and lung cancers [125]. The main risks for cancer patients resulting from the development of cachexia include the following:

- Body weakness—loss of muscle mass impairs function and reduces the patient’s quality of life.
- Increased susceptibility to infections—malnutrition weakens the immune system, which increases the risk of complications.
- Decreased therapy effectiveness—cachectic patients have a poorer tolerance to chemotherapy and radiotherapy, which may lead to treatment limitations.Although IF can regulate cancer cell metabolism, its use in patients with advanced cancer remains controversial. The lack of conclusive clinical evidence suggesting benefits of this strategy in patients with cachexia means that further research is needed [7,126].

## 4. Conclusions

Intermittent fasting (IF) and the regulation of autophagy are promising strategies to support cancer treatment, influencing key metabolic and signaling pathways. IF, by inhibiting the mTOR pathway and activating AMPK, induces autophagy, which can both limit cancer cell proliferation and enhance the effectiveness of chemotherapy and radiotherapy. Additionally, changes in the tumor microenvironment, such as reduced polarization of M2 macrophages, decreased angiogenesis, and limited nutrient availability, further contribute to the potential effectiveness of IF in oncological therapy. Despite numerous preclinical findings, the clinical application of IF in cancer treatment still requires additional research. Significant challenges remain, such as individual patient responses to IF, the risk of malnutrition, and the lack of standardized fasting protocols tailored to different cancer types. Well-designed clinical studies are needed to assess the long-term efficacy and safety of IF and to determine optimal fasting regimens in combination with cancer therapies.

In summary, IF has the potential to support cancer treatment by affecting cellular metabolism, autophagy, and the tumor immune response. However, there is no universal dietary model suitable for all patients, and further research is needed to determine effective dietary regimens to support oncological therapy.

## 5. Materials and Methods

### Literature Search Strategy

A comprehensive literature search was performed using the PubMed database, without date restrictions, to identify relevant studies investigating the relationship between intermittent fasting, autophagy, and cancer. The search was conducted using a combination of Medical Subject Headings (MeSH) and free-text terms in English.

No filters were applied regarding the article type in order to include both original research articles and reviews.

The search strategy included the following queries:

- (autophagy[Title/Abstract] OR “Autophagy”[MeSH Terms]) AND (intermittent fasting[Title/Abstract] OR “Intermittent Fasting”[MeSH Terms]) AND (cancer[Title/Abstract] OR tumor[Title/Abstract] OR neoplasm[Title/Abstract])
- (autophagy[Title/Abstract]) AND (fasting mimicking diet[Title/Abstract]) AND (cancer[Title/Abstract])
- (autophagy[Title/Abstract]) AND (caloric restriction[Title/Abstract]) AND (neoplasm[Title/Abstract])
- (intermittent fasting[Title/Abstract]) AND (tumor metabolism[Title/Abstract]) AND (autophagy[Title/Abstract])
- (fasting[Title/Abstract]) AND (chemosensitization[Title/Abstract]) AND (autophagy[Title/Abstract]) AND (cancer[Title/Abstract])

## Figures and Tables

**Table 1:** Studies on humans showing the effects of dietary interventions in combination with conventional therapies in the context of cancer diseases.

Cancer Type	Primary Therapy Strategy	Diet Type	Therapeutic Effect	Study Population	Number of Participants	Study
various types	various standard types—chemotherapy, radiotherapy, surgery	intermittent fasting (2 days per week)	reduction in IGF-1 levels, improved quality of life	patients with multiple cancer types	10	[6]
breast cancer	no therapy before surgery	fasting-mimicking diet (FMD) for 5 days per month	reduction in glucose and insulin levels, potential autophagy activation	women with breast cancer in the preoperative period	34	[119]
glioma	radiotherapy	intermittent fasting (50% calorie restriction every other day)	disease stabilization, improved response to radiotherapy	patients with glioma	8	[120]
gynecological cancers	chemotherapy—carboplatin, paclitaxel	intermittent fasting (16:8)	improved well-being and reduction in side effects of oncological therapy	women with gynecological cancers	47	[121]
glioma	radiotherapy; chemotherapy—temozolomide	ketogenic diet/intermittent fasting	in some cases, slowed tumor progression	patients with glioma	25	[122]
various cancer types	various standard treatments	caloric restriction and time-restricted feeding (TRF)	lowered blood glucose (by 18.6%), insulin (by 50.7%), and IGF-1 (by 30.3%); increased activation of CD8+ T cells	patients with multiple cancer types	101	[123]

## Abbreviations

The following abbreviations are used in this manuscript:

3-MA	3-methyladenine
Akt	protein kinase B (PKB)
Ambra1	autophagy and Beclin 1 regulator 1
AMPK	5′AMP-activated protein kinase
ATG5/7/12	autophagy gene 5/7/12
Bcl2	B-cell lymphoma 2
BECN2	Beclin 2
BECN1	Beclin 1
BPC11	biphosphinic paladacycle complex 11
BRAF	B-Raf proto-oncogene, serine/threonine kinase
cAMP	cyclic adenosine monophosphate
CMA	chaperone-mediated autophagy
CR	caloric restriction
CRP	C-reactive protein
CSCs	cancer stem cells
FMD	fasting-mimicking diet
GLUT1/2	glucose transporter ½
HDAC	histone deacetylase
HDL	high-density lipoprotein
HIF-1α	hypoxia-inducible factor 1-alpha
IF	intermittent fasting
IGF-1	insulin-like growth factor 1
JAK1/STAT	Janus kinase 1/signal transducer and activator of transcription
LAMP2A	lysosome-associated membrane protein 2A
LDL	low-density lipoprotein
MCL1	myeloid cell leukemia 1
MDM2	mouse double minute 2 homolog
MDSC	myeloid-derived suppressor cells
MHC II	major histocompatibility complex class II
mTOR	mammalian target of rapamycin
mTORC1	mammalian target of rapamycin complex 1
MYC	MYC proto-oncogene, bHLH transcription factor
NAFLD	non-alcoholic fatty liver disease
NGFR	nerve growth factor receptor
NF-κB	nuclear factor kappa-light-chain-enhancer of activated B cells
NK-cells	natural killer cells
PF	periodic fasting
p53	tumor protein p53
PI3K	phosphoinositide 3-kinase
PINK1	PTEN-induced kinase 1
PKA	protein kinase A
RND3	Rho family GTPase 3 (RhoE)
ROS	reactive oxygen species
TAM	tumor-associated macrophage
TFEB	transcription factor EB
TNF-α	tumor necrosis factor alpha
TPT1	tumor protein, translationally controlled 1
Tregs	regulatory T cells
TRF	time-restricted feeding
ULK1	Unc-51-like autophagy activating kinase 1
UCP1	uncoupling protein 1
VEGF	vascular endothelial growth factor
WHO	World Health Organization
